# Algorithmic considerations when analysing capture Hi-C data

**DOI:** 10.12688/wellcomeopenres.16394.1

**Published:** 2020-12-14

**Authors:** Linden Disney-Hogg, Ben Kinnersley, Richard Houlston

**Affiliations:** 1 Division of Genetics and Epidemiology, The Institute of Cancer Research and The Royal Marsden NHS Foundation Trust, Sutton, Surrey, SM2 5NG, UK; 2 Present address: School of Mathematics, University of Edinburgh, Edinburgh, EH9 3FD, UK

**Keywords:** Capture Hi-C, Model assessment, Cancer

## Abstract

Chromosome conformation capture methodologies have provided insight into the effect of 3D genomic architecture on gene regulation. Capture Hi-C (CHi-C) is a recent extension of Hi-C that improves the effective resolution of chromatin interactions by enriching for defined regions of biological relevance. The varying targeting efficiency between capture regions, however, introduces bias not present in conventional Hi-C, making analysis more complicated. Here we consider salient features of an algorithm that should be considered in evaluating the performance of a program used to analyse CHi-C data in order to infer meaningful interactions. We use the program CHICAGO to analyse promotor capture Hi-C data generated on 28 different cell lines as a case study.

## Introduction

**Figure 1.**
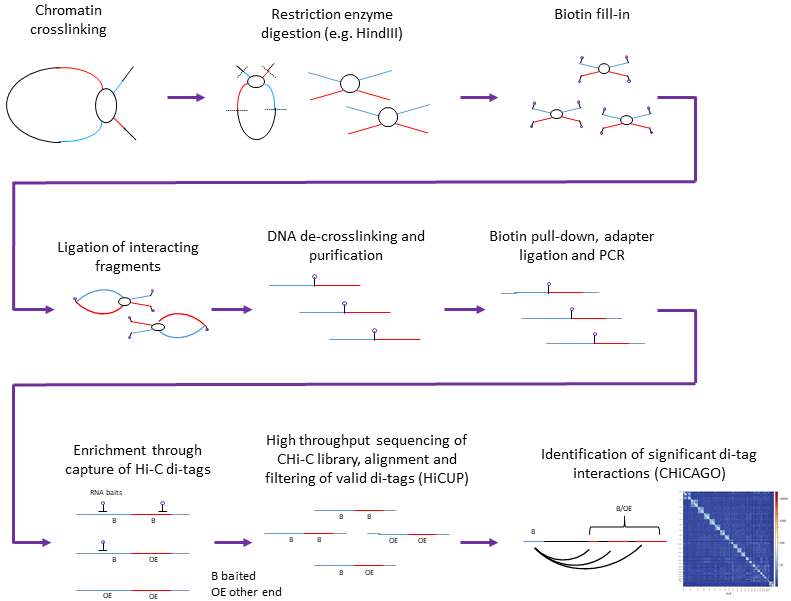
Work-flow for the generation of CHi-C library and downstream analysis. Herein we consider data based on restriction enzyme digestion using the 6bp cutter Hind III, Sequencing of Chi-C library, alignment and filtering of valid di-tags using HiCUP ^9^ and identification of significant di-tag interactions using CHiCAGO ^8^.

Chromosome conformation capture (3C) methodologies ^1– 3^ have provided insight into the effect of 3D genomic architecture on gene regulation ^4– 6^. They preserve chromatin interactions by cross-linking followed by fragmentation, ligation and sequencing of interacting genomic regions. Hi-C exploits high-throughput paired-end sequencing to retrieve a short sequence from each end of each ligated fragment, allowing all pairwise interactions between fragments to be tested ^7^ ( Figure 1).

Chromatin interactions can result from biological functions, such as promoter-enhancer interactions, or from random polymer looping, whereby undirected physical motion of chromatin causes loci to collide. To identify ‘true’ interactions, it is necessary to identify the contribution from the null hypothesis, largely attributed to constrained Brownian motion and noise ^8^. While not completely eliminating background noise, the development of *in situ* Hi-C, which preserves the integrity of the nucleus during Hi-C library generation, has gone some way to reducing it ^3^.

Analysis of Hi-C libraries involves filtering of invalid di-tags such as self-ligated pairs or adjacent fragment di-tags ^9^ before determining statistically significant and biologically important di-tag interactions. The expected frequency of interactions between two fragments decreases with their genomic distance, especially if the fragments lie in different chromosomes ^8^. Hence, reliable estimates of the dependence on distance are a prerequisite to any analysis.

While Hi-C allows for genome-wide characterization of chromatin contacts detection its effective resolution is determined by both restriction fragmentation and sensitivity of the experiment. Capture Hi-C (CHi-C) is a recent extension of the Hi-C methodology that improves resolution by enriching defined regions of biological significance ^10^ ( Figure 1). Analysis of CHi-C data is, however, more complicated than conventional Hi-C because: (1) varying targeting efficiency between capture regions introduces a bias not present in Hi-C ^8^; (2) contact maps in CHi-C arise from two distinct sources that have innately different visibility profiles - between the two captured fragments and between captured and non-captured fragments; (3) null hypotheses for each di-tag pair are not independent as these are tested simultaneously, requiring an alternative statistic instead of reliance on raw *p*-values from hypothesis tests. 

CHi-C, especially in the guise of promotor capture Hi-C (PCHi-C) is increasingly being used to decipher the genetic basis of aberrant gene expression in cancer. Since cancers rarely have diploid genomes, the analysis of PCHi-C from tumours is further complicated by copy number variation (CNV) ^11^ and presence of inter-chromosomal translocations ^12^.

Here we examine a number of features of an algorithm that should be considered in evaluating the performance of a program used to analyse CHi-C data. Specifically, (i) the appropriateness of the distance-correction used in the model; (ii) relative importance of weights assigned to model parameters; (iii) whether the null is accurately reproducing the distribution of the large majority of contacts and how thresholds for declaring significant interactions are obtained; (iv) whether the underlying model leads to asymmetry in test statistics of bait-bait pairs; and (v) how an algorithm behaves processing CHi-C cancer cell line data. As an illustration we consider CHiCAGO ^8^ as a case study since it is a widely-used program for analysing PCHi-C data ^13, 14^. The algorithm features a novel background correction procedure using a two-component convolution model designed to account for real but expected interactions as well as experimental and sequence-based artefacts. Additionally, CHiCAGO implements a *p*-value weighting procedure, based on parameters that can be estimated from the data.

## Results

### Cell lines and PCHi-C data

We made use of publicly accessible PCHi-C cell line data - 18 human primary hematopoietic cells (lymphoblastoid - GM12878; activated and non-activated total CD4+ - ACD4 and NACD4, respectively; endothelial precursors – EP; erythroblasts - Ery; foetal thymus – FoeT; macrophage - Mac0, Mac1, Mac2; megakaryocytes – MK; monocytes – Mon; naive and total B, CD4+, CD8+ – NB, NCD4, NCD8, TB, TCD4 and TCD8 respectively; and neutrophils – Neu) and 10 cancer cell lines (glioma - BLN2, BLN3, N16; colorectal cancer - HT29, LoVo; multiple myeloma - KMS11, KM12BM, MM1S; Hodgkin lymphoma - L428; testicular germ cell tumor - NTERA2); detailed in Supplementary Table 1 (see *Extended data* ^15^).

Raw sequencing data was processed using HiCUP v0.6.1 ^9^ to obtain only valid interaction di-tags aligned to build 38 of the human genome **.**Summary statistics for each PCHi-C dataset are provided in Supplementary Data 1 (see *Extended data* ^15^). Significance of interaction frequencies for di-tags, both ends baited (bait-bait) and where only one end was baited (bait-other end) were estimated using CHiCAGO v1.1.8 ^8^.

### Model considerations

We initially considered PCHi-C libraries from the 18 non-tumour cell lines. In CHiCAGO, background interactions are modelled by the following components of a Delaporte distribution, which are assumed to be independent: (1) Brownian collisions – modelled by a negative binomial random variable with expected levels a function of genomic distance, adjustment for biases associated with individual fragments and size parameter independent of the interacting pair; (2) assay artefacts/technical noise ( *i.e*. sequencing errors) – modelled by a Poisson random variable, whereby the mean of Poisson random variable depends on the properties of interacting fragments, but is independent of genomic distance between fragments.

We examined the validity of the model and estimation of central parameters. Assuming that in ‘small’ distance bins technical noise is low, as per CHiCAGO specifications, test statistics and corresponding *p*-values for the Kolmogorov-Smirnov (KS) test (testing probability that data observed is generated by the model specified, aggregated over distance bins) were generated for ACD4 ( Table 1) and the other 17 cell lines (Supplementary Table 2, *Extended data* ^15^). In most cell lines the *p*-values associated with the small distance bins were effectively zero but rapidly increased to near-unity in the larger distance bins. The notable outlier was GM12878, which was typified by near zero *p*-values across all distance bins. The exact estimates of bin-wise *p*-values are not necessarily important, since they will be impacted by true interactions, bait specific biases permitted by the model, the effect of which is expected to be small, and the distribution of distances within each bin. Nevertheless, the fact that there was no rejection of the null hypothesis at large distance bins implies that the negative binomial model fits the data well for broad-scale behaviour. As technical noise is proportionally greater at large distances, the discrepancy in how well the negative binomial fits over distance cannot be attributed to the KS test ignoring the Poisson component.

**Table 1.** Discrete KS test applied to the null distribution for ACD4. Bin-wise D statistics and the corresponding Monte Carlo *p*-value for the ACD4 cell line.

Distance Bin	Test Statistic	P value
(0,2e+04]	0.228	<0.001
(2e+04,4e+04]	0.208	<0.001
(4e+04,6e+04]	0.191	<0.001
(6e+04,8e+04]	0.180	<0.001
(8e+04,1e+05]	0.172	<0.001
(1e+05,1.2e+05]	0.166	<0.001
(1.2e+05,1.4e+05]	0.162	<0.001
(1.4e+05,1.6e+05]	0.155	<0.001
(1.6e+05,1.8e+05]	0.148	<0.001
(1.8e+05,2e+05]	0.142	<0.001
(2e+05,2.2e+05]	0.137	<0.001
(2.2e+05,2.4e+05]	0.130	<0.001
(2.4e+05,2.6e+05]	0.124	<0.001
(2.6e+05,2.8e+05]	0.118	<0.001
(2.8e+05,3e+05]	0.113	<0.001
(3e+05,3.2e+05]	0.107	<0.001
(3.2e+05,3.4e+05]	0.100	<0.001
(3.4e+05,3.6e+05]	0.095	<0.001
(3.6e+05,3.8e+05]	0.091	<0.001
(3.8e+05,4e+05]	0.086	<0.001
(4e+05,4.2e+05]	0.078	<0.001
(4.2e+05,4.4e+05]	0.078	<0.001
(4.4e+05,4.6e+05]	0.079	<0.001
(4.6e+05,4.8e+05]	0.083	<0.001
(4.8e+05,5e+05]	0.084	<0.001
(5e+05,5.2e+05]	0.086	<0.001
(5.2e+05,5.4e+05]	0.090	<0.001
(5.4e+05,5.6e+05]	0.089	<0.001
(5.6e+05,5.8e+05]	0.090	<0.001
(5.8e+05,6e+05]	0.091	<0.001
(6e+05,6.2e+05]	0.093	<0.001
(6.2e+05,6.4e+05]	0.094	<0.001
(6.4e+05,6.6e+05]	0.098	<0.001
(6.6e+05,6.8e+05]	0.100	<0.001
(6.8e+05,7e+05]	0.102	<0.001
(7e+05,7.2e+05]	0.101	<0.001
(7.2e+05,7.4e+05]	0.105	<0.001
(7.4e+05,7.6e+05]	0.106	<0.001
(7.6e+05,7.8e+05]	0.112	<0.001
(7.8e+05,8e+05]	0.111	<0.001
(8e+05,8.2e+05]	0.113	<0.001
(8.2e+05,8.4e+05]	0.115	<0.001
(8.4e+05,8.6e+05]	0.114	<0.001
(8.6e+05,8.8e+05]	0.118	<0.001
(8.8e+05,9e+05]	0.119	<0.001
(9e+05,9.2e+05]	0.121	<0.001
(9.2e+05,9.4e+05]	0.122	0.001
(9.4e+05,9.6e+05]	0.124	0.012
(9.6e+05,9.8e+05]	0.126	0.478
(9.8e+05,1e+06]	0.126	>0.999
(1e+06,1.02e+06]	0.129	>0.999
(1.02e+06,1.04e+06]	0.129	>0.999
(1.04e+06,1.06e+06]	0.131	>0.999
(1.06e+06,1.08e+06]	0.131	>0.999
(1.08e+06,1.1e+06]	0.134	>0.999
(1.1e+06,1.12e+06]	0.135	>0.999
(1.12e+06,1.14e+06]	0.136	>0.999
(1.14e+06,1.16e+06]	0.137	>0.999
(1.16e+06,1.18e+06]	0.138	>0.999
(1.18e+06,1.2e+06]	0.141	>0.999
(1.2e+06,1.22e+06]	0.143	>0.999
(1.22e+06,1.24e+06]	0.143	>0.999
(1.24e+06,1.26e+06]	0.143	>0.999
(1.26e+06,1.28e+06]	0.147	>0.999
(1.28e+06,1.3e+06]	0.148	>0.999
(1.3e+06,1.32e+06]	0.150	>0.999
(1.32e+06,1.34e+06]	0.149	>0.999
(1.34e+06,1.36e+06]	0.149	>0.999
(1.36e+06,1.38e+06]	0.152	>0.999
(1.38e+06,1.4e+06]	0.154	>0.999
(1.4e+06,1.42e+06]	0.154	>0.999
(1.42e+06,1.44e+06]	0.155	>0.999
(1.44e+06,1.46e+06]	0.157	>0.999
(1.46e+06,1.48e+06]	0.158	>0.999
(1.48e+06,1.5e+06]	0.160	>0.999

The ‘distance function’, a key component of CHiCAGO’s implementation of a genomic distance dependence into the mean of the negative binomial, was generated for each cell line. Coefficients of distance fit curves and plots of estimates of the distance function *f*( *d*) for the 18 cell lines are shown in Supplementary Table 3 and Supplementary Figure 1 (see *Extended data* ^15^). In all 18 cases the cubic spline fitted by CHiCAGO provides a good fit to the data. With the exception of GM12878, there was strong concordance between the theoretical, linear and cubic fit, and the curvature of the cubic spline *K* further shows GM12878 as an outlier, which may well reflect GM12878 Hi-C libraries being prepared by dilution rather than *in situ* ligation. In view of GM12878 being an outlier, a linear model *Linear Intercept ~ Linear Gradient* was fitted with and without GM12878 ( *y* = – *0.776* – *14.681x* and *y* = *1.067* – *12.919x*, respectively), with the second having a lower residual square sum (RSS), so correspondingly providing for a better fit. This linear fit is consistent with interactions detected primarily being cis-chromosomal.

The assumption that assay artefacts have minimal effect on expected reads in small distance bins was confirmed by calculating the mean technical noise parameter *λ* and mean number of trans pairs observed per bait for each cell line. Box plots of the parameter estimate per bait or other-end pool, shown in Supplementary Figure 2 (see *Extended data* ^15^), adhere largely to the patterns expected as laid out in the CHiCAGO vignette ^8^, where it is stated that to interpret the noise box plots one needs to check that “distributions’ median and variance should trend upwards as we move from left to right”.

### Score statistic

**Figure 2.**
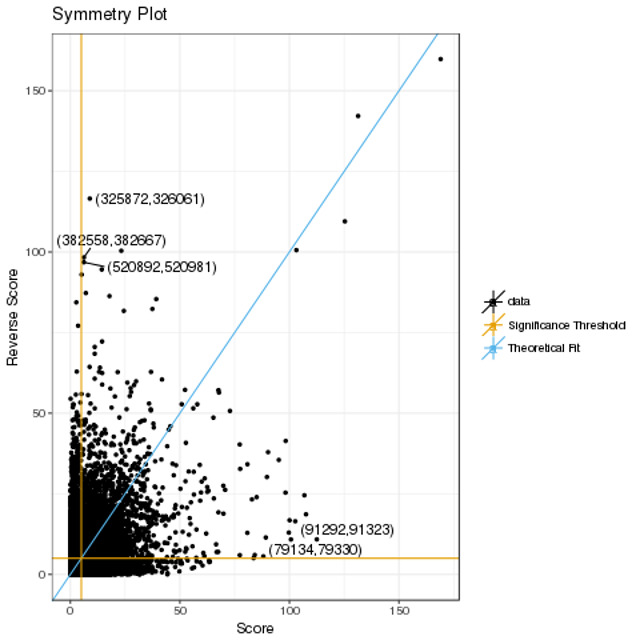
Plots of the score statistics for bait-bait pairs in ACD4. The five pairs with the largest deviation, (Score – Reverse Score) ^2^, are labelled with their IDs from the rmap file. The significance threshold of 5 is indicated by orange lines, with the blue line corresponding to the theoretical fit of Score=Reverse Score.

CHiCAGO implements a novel score statistic as a proxy for the strength of evidence supporting an interaction ^8^. We investigated the suitability of this statistic and the threshold advocated for declaring significance. Initially, we compared CHiCAGO interaction scores between bait-bait pairs. Intuitively it might be assumed that the score for baits AB will be identical to BA. However, this is not the case as evidenced by plots of *score _ij_* against *score _ji_* statistics for ACD4 ( Figure 2) and the other 17 cell lines (Supplementary Figure 3). The asymmetry arises because when CHiCAGO constructs bait-end biases and other end biases, the former are assumed to be fixed for each bait, whereas the other-end bias is assumed to be drawn from a random distribution, resulting in a different number of expected reads for the pair (mean correlation 0.4854, interquartile range (IQR) 0.0970). To further understand this asymmetry, we define an interaction as ‘reversible’ if, for a given threshold, the significance of the interaction did not depend on the direction in which the score was calculated. The mean percentage of reversible interactions was only 23.06% (IQR = 4.06%); the presence of non-reversible interactions representing failure of the algorithm to assign biological relevance to a bait-bait interaction.

### Significance threshold

The threshold advocated by the developers of CHiCAGO for declaring a significant interaction is a *score*_*ij*_ > 5 ^8^ - referred to as the normal score threshold (NST). We investigated power and false-discovery rate (FDR) at this threshold. To evaluate power, or equivalently the false-negative rate, (FNR) we calculated the proportion of interactions with log( *p*) < – *10* (considering the null hypothesis of the Delaporte distribution) and score >5. log( *p*) < – *10* ( *i.e.* ‘robust’ interactions) was used as in the mathematical specification of CHiCAGO it is suggested that reproducible interactions are those that pass this threshold in all replicates ^8^.

Because the principle underlying the CHiCAGO score statistic precluded simply using *p-*values to identify true interactions, except for in extreme cases, we used the Jaccard index as a proxy for FDR (assuming interactions passing the score threshold in all replicates are true). To quantify the suitability of the score as a statistic at alternate thresholds, we calculated alternate score thresholds with the aim of improving the FNR, FDR, as well as the family-wise error rate (FWER) ( Table 2). Across all 18 cell lines the threshold to fix the theoretical FWER was a score ≳ *15*; however, this limited discovery to *O*( *10 ^4^*) interactions per cell line, compared with *O*( *10 ^5^*) interactions when imposing a score threshold of 5.

**Table 2.** Suggested score thresholds (SSTs) and corresponding quality summary statistics. SSTs found seeking to fix or optimise certain quality statistics of the data. All SSTs are given to the nearest integer.

Cell	SST giving FNR=0.2	Achieved FDR (at NST) (2.d.p)	SST minimising FDR (using BFGS)	Achieved FDR (using BFGS) (2.d.p)	SST minimising FDR (using NM)	Achieved FDR (using NM) (2.d.p)	SST giving FWER=0.05
ACD4	4	0.84	5	0.84	502	0.00	15
EP	10	0.83	5	0.83	1468	1.00	15
Ery	10	0.89	5	0.89	2741	1.00	15
FoeT	2	0.85	27	0.78	452	0.00	15
GM12878	10	0.97	3	0.97	122	0.00	15
Mac0	9	0.86	5	0.85	3761	1.00	15
Mac1	10	0.86	5	0.86	559	0.33	15
Mac2	10	0.87	5	0.87	1396	1.00	15
MK	11	0.87	5	0.87	1805	1.00	15
Mon	8	0.86	5	0.86	1200	1.00	15
NACD4	4	0.85	5	0.85	1412	1.00	15
naiveB	8	0.85	6	0.84	447	0.50	15
NCD4	8	0.90	5	0.90	1882	1.00	15
NCD8	5	0.85	5	0.85	2268	1.00	15
Neu	5	0.90	5	0.90	1108	1.00	15
TB	5	0.86	30	0.79	576	0.00	15
TCD4	4	0.88	5	0.88	2314	1.00	15
TCD8	6	0.84	19	0.78	1367	1.00	15

SST, suggested score threshold; FNR, false-negative rate; FDR, false-discovery rate; NST, normal score threshold; BFGS, Broyden–Fletcher–Goldfarb–Shanno; NM, Nelder-Mead; FWER, family-wise error rate.

### Weighting procedure

CHiCAGO scores are computed from raw *p*-values corrected for the prior probability of true interaction, fitted by a four-parameter logistic regression model ^8^. The parameters can be calculated from the reproducibility of interaction frequencies at different genomic distances for the cell line, otherwise by default the program uses estimates from macrophage data. To investigate the appropriateness of the estimation method and the extent to which the choice of weighting affects the identification of significant interactions, we used CHiCAGO’s pre-built method to calculate parameters for each cell line.

CHiCAGO uses the observed interactions to fit a curve of true-interaction prior probability that decreases monotonically with distance. The monotonicity of the model is intuitive because in general baits that have a greater separation distance will have a lower prior probability of interacting. The lack of the monotonicity of the observed data, a measure with range of 0 to 1, with 0 corresponding to perfect monotonicity, had mean 0.3222, IQR 0.0917 (4.d.p). While it is not possible to quantify how much of the lack of monotonicity is due to expected variance without making additional hypotheses of the model, visual inspection of the data the weighting curves produced by CHiCAGO fit to, as shown in Supplementary Figure 4 (see *Extended data* ^15^), shows a local peak around a log distance of 12. This behaviour is not detectable by the logistic model which is monotonically decreasing. This suggests that the non-zero lack of monotonicity is caused by underlying biological features.

The RSS for the logistic regression had mean 26.08, IQR 6.50, the GM12878 cell line an outlier with RSS 73.11. Visually this is identifiable as the fit is near horizontal for the GM12878 cell line. GM12878 in fact has a lower RSS than one would expect, as there were only seven distance bins contributing, all others having zero observed interactions. This is a more general phenomenon whereby fits in which there are zero-observed-interaction bins will have an artificially lower RSS.

As the threshold of log( *p*) < – *10* for defining ‘true’ interactions is somewhat arbitrary, yet it affects weight parameters, we sought an alternate *p*-threshold and recalculated the RSS. With the weight parameters calculated by CHiCAGO that minimised the RSS, key statistics for the data were recalculated, giving a mean change of 0.0153 for the score correlation, 1.36% for the reversibility, 0.0355 for the FDR at the NST, and -0.0708 for the FNR. This suggests that, on balance, using the custom calculated weight parameters improves the quality of the resulting calculations. Moreover, the Jaccard index for concordance between significant interactions with and without suggested weights had mean and IQR values of 0.8732 and 0.0737, respectively, demonstrating that the choice of weights affects which interactions are reported as significant. Applying the new weights had no significant effect on the number of interactions observed at the NST.

### Application to cancer cell lines

We next analysed PCHi-C data generated on the 10 cancer cell lines. Supplementary Table 4 and Supplementary Figure 5 (see *Extended data* ^15^) show coefficients and plots of distance curve fits for these cell lines. In contrast to the non-cancer cell lines where the linear fit to the distance curve was essentially concordant with the data, this was not the case for cancer cell lines. Noticeably, in all the cancer cell lines the cubic fit had a negative cubic coefficient, which corresponds physically to observing a larger number of interactions at middling distances ( *O*(10 ^5^) bps). This could be the result of small-scale translocations, which would have the same effect on the expected number of reads. While deviation from linear of their cubic splines had a mean curvature of 0.2685 compared to 0.1149, the corresponding fit of the linear intercept to gradient was *y* = *1.107 – 13.000x* with an RSS of 0.189, similar to the non-cancer cell lines and showing the same consistency, with almost all interactions detected being cis-chromosomal.

Plots of the score symmetry for bait-bait pairs are shown in Supplementary Figure 6 (see *Extended data* ^15^). Cancer cell lines tended to have a higher non-zero score correlation for bait-bait pairs (mean 0.5136, IQR 0.1803) but a significantly lower percentage of reversible interactions (mean 14.54%, IQR 6.54%). The BLN2 and BLN3 cell lines showed substantive aberrant behaviour in their plots in which proximal bait-bait pairs in a similar region extended in long ‘arms’ away from the theoretical fit. This was not seen in any of the other cell lines and is likely to be a consequence of a vastly different underlying genomic architecture. Summary statistics to calculate the quality of the score threshold were again calculated. The FNR was higher for cancer cell lines (mean 0.197, IQR 0.069). Suggested score thresholds to improve the FNR or theoretical FWER ( Table 3), were similar to those seen for non-cancer cell lines. 

**Table 3.** Suggested score thresholds (SSTs) and corresponding quality summary statistics for cancer cell lines. SSTs found seeking to fix or optimise certain quality statistics of the data. All SSTs are given to the nearest integer.

Cell	SST giving FNR=0.2	Achieved FDR (at NST) (2.d.p)	SST minimising FDR (using BFGS)	Achieved FDR (using BFGS) (2.d.p)	SST minimising FDR (using NM)	Achieved FDR (using NM) (2.d.p)	SST giving FWER=0.05
BLN2	2	0.74	22	0.61	390	0.48	15
BLN3	5	0.75	5	0.78	432	0.88	15
HT29	4	0.89	5	0.89	116	0.58	15
KMS11	5	0.85	71	0.83	86	0.82	15
KMS12BM	7	0.84	5	0.84	898	0.29	15
L428	9	0.78	5	0.78	904	0.57	15
LoVo	10	0.89	5	0.89	212	0.75	15
MM1S	7	0.85	5	0.85	238	0.43	15
N16	3	0.81	5	0.81	138	0.5	15
NT2	7	0.70	5	0.70	12	0.68	15

SST, suggested score threshold; FNR, false-negative rate; FDR, false-discovery rate; NST, normal score threshold; BFGS, Broyden–Fletcher–Goldfarb–Shanno; NM, Nelder-Mead; FWER, family-wise error rate.

The data showed a lack of the monotonicity (mean 0.5999, IQR 0.0865 (4.d.p); mean RSS of 55.4, IQR 56.9), but was significantly higher than that observed in non-cancer cell lines. This distortion was most pronounced with BLN2, BLN3 and HT29 cell lines, which all showed very low concordance between the fit and data points. Mean changes in summary statistics with CHiCAGO-calculated weight parameters were 0.0292 for score correlation, 2.27% for reversibility, 0.0683 for FDR at the NST and -0.0870 for the FNR. The corresponding mean Jaccard index was 0.6878 (IQR, 0.2010), highlighting the importance of using derived weights in analyses.

Finally, we examined heatmaps of Hi-C interaction frequencies to detect potential cancer-related chromosomal abnormalities, finding that BLN2 and BLN3 exhibit large-scale inter-chromosomal translocations (Supplementary Figure 7, *Extended data* ^15^) **.**However, such features are unlikely to be sufficient to solely account for the increased score asymmetry observed in cancer cell lines.

## Discussion

When utilising any statistical test, it is necessary to verify that any necessary properties of input data are satisfied, and that under these assumptions sensible conclusions are drawn. In this study we have sought to evaluate CHiCAGO as a methodology for identifying statistically significant genomic interactions in PCHi-C data. This evaluation included examination of: (i) the suitability of the distance-correction model employed; (ii) evidence of discordance in association statistics at bait-bait pairs; (iii) significance thresholds of called interactions; (iv) importance of weight parameter estimates; (v) specific considerations for its application to analysis of cancer cell-line data.

Our findings indicate that the Delaporte null fitted the data well in large distance bins, with the assumption that the Poisson contribution is small being verified. The cubic spline distance function fitted the data well, with the linear fit being sufficient for most non-cancerous cell lines. The symmetry in the score parameter was very low for bait-bait pairs. The default CHiCAGO score threshold of >5 was typically too low to ensure reliability in the data, evaluated either from the FNR or FWER, but correspondingly the sensitivity to detect interactions was greater than at higher score thresholds, with a resulting higher false discovery rate. Using custom cell-line specific weight parameters marginally improved summary statistics of the data compared to reliance on default parameters. The overlap of significant interactions with and without suggested parameters was around 90%, demonstrating the presence of either false positives or false negatives when using standard weight parameters. These features were also seen, albeit more pronounced, in cancer cell lines. Using custom weights improved the metrics applied to the output, as expected since it provides the theory-mandated adjustment of the *p*-values.

As the framework we provide only considers how CHiCAGO processes input data, our methodology is largely resistant to limitations due to the underlying CHi-C inputs. There are small differences in the two versions of the designed oligonucleotide baits to capture promoter fragments between cancer and non-cancer cell line data, but as CHiCAGO produces bait-specific biases as part of its model, we should not expect this to have a major influence on our conclusions. For calculations involving the FDR, FNR, and FWER, due to the lack of *bona fide* reference interactions, we were reliant on theoretically equivalent proxies. As a result, point estimates will be inherently imprecise and allow us to either only make comparisons or reference confidence intervals between different score thresholds. Furthermore, although we provide a large range of statistics as an example of how to assess Hi-C algorithms, there are some visual features that are not necessarily amenable to numerical description. Criteria for selecting the bests summary statistics to efficiently assess algorithms is desirable, something that may potentially be tractable by applying approximate Bayesian computation ^16^. 

From a numerical and computational perspective our study highlights a few key points. The fact that the cubic distance function fit implemented by CHiCAGO correctly matched the data for every cell line is unsurprising, given the large number of parameters it was able to utilise. We should similarly expect the same of the logistic regression, and so large failures to fit the data are indicative of the unsuitability of the underlying form of the curve for the data it is approximating. Moreover, the exact implementation of methodologies is demonstrated to be important. Numerical optimisation improved the FDR on average, but Nelder-Mead (NM) often produced thresholds too large to be useful in discovery, serving to demonstrate the importance of understanding the underlying processes behind standard R functions, as Broyden–Fletcher–Goldfarb–Shanno (BFGS) provided more practical thresholds. This difference in behaviour stems both from the fact that the Jaccard index is not a continuous function of the score threshold, and that NM is a heuristic algorithm.

Inevitably, a challenge in evaluating the performance of Hi-C and Hi-C algorithms is not having reference to a large “gold standard” reference set of *bona fide* true interactions in a given cell line. Forcato *et al.*, 2017 ^17^ have detailed experimental validations from the literature of a set of Hi-C interactions using 3C or fluorescence *in situ* hybridisation (FISH) in a series of cell lines. Given 3C is essentially analogous to Hi-C, it can be questioned if such data is truly orthogonal. Additionally, FISH is limited to examination of long-range interactions. In the absence of experimental data, it has been proposed that demonstrating significant contacts are enriched for biologically relevant regulatory features ( *e.g.* open chromatin, transcriptional activation) provides validation. While this provides validation of experimental enrichment of for example promoters, it does not, however, necessarily follow that the identified interactions are *bona fide*. Javierre *et al.*, 2016 ^13^, have proposed generating and sequencing “reverse PCHi-C” libraries, designing baits to the unbaited ends of previously identified interactions, to determine if previously identified interactions are recovered. One way of generating a “null model” for comparison of di-tag interaction frequencies is to sequence a generated “random ligation” library prepared by reversal of cross-links prior to ligation ^10^. This has, however, not generally been standard practise in preparation of large numbers of libraries.

Analysis of CHi-C data generated from cancer cells clearly presents challenges beyond that of diploid cells. Translocations affect distance estimates, leading to highly significant interaction *p*-values between translocation breakpoints. As a prelude to any analysis of CHi-C, examining pre-capture Hi-C data can be used to identify translocations and inform downstream analyses. Other molecular abnormalities in cancer cell lines, such as focal amplifications/deletions, regions of kataegis and chromothripsis are more intractable sources of bias. Comprehensively accounting for such aberrations ideally requires *de novo* assembly of the cancer genome being investigated.

In conclusion, our analysis highlights a number of features that should be considered when evaluating CHi-C algorithms. In application to CHiCAGO, while we saw that the underlying null hypothesis was entirely sensible, assigning significance to a given interaction is not entirely straightforward. It is clear that many issues associated with processing of CHi-C data are exacerbated when studying cancer derived data because of the complex nature of their genomes.

## Methods

### Datasets analysed

The 28 cell lines and PCHi-C datasets analysed are detailed in Supplementary Table 1 (see *Extended data* ^15^), see also *Underlying data*.

### Extracting valid PCHi-C interacting fragments and identification of interactions

HiCUP v0.6.1 ^9^ was used to map reads to human build 38 using bowtie v2.3.4 ^18^, pair reads and filter valid interaction di-tags. CHiCAGO v1.1.8 ^8^ was used to estimate significance of interaction frequencies between restriction fragment di-tags using the appropriate baitmap file (Supplementary Table 1, *Extended data* ^15^). Read counts for PCHi-C datasets at each stage of HiCUP processing are detailed in Supplementary Data 1 (see *Extended data* ^15^). Genome-wide heatmaps of Hi-C contacts were generated using HiCExplorer v2.1.1 ^19^ to identify large-scale chromosomal translocations.

### Evaluation of CHiCAGO

Cairns *et al*. ^8^ provide a mathematical specification of the algorithm used by CHiCAGO, and we utilise the same notation. For pairs less than 1.5 Megabasepairs (Mbp) apart, the CHiCAGO algorithm assumes that the contribution to the total number of counts from the ‘technical noise’ component of the null model employed is sufficiently lower than that from the ‘Brownian’ component, so it is reasonable to approximate the model as


$$X_{ij}∼NB(\mu_{ij},r),$$

where *NB*( *μ*, *r*) is a negative binomial distribution with probability mass function


$$f_{NB}(k;\mu ,r)=\frac{\Gamma (r+k)}{k!\Gamma (r)}{\left(\frac{\mu }{r+\mu }\right)}^{k}{\left(\frac{r}{r+\mu }\right)}^{r},$$

$$k\in N_{0};\mu ,r\in R_{>0}.$$

Discrete Kolmogorov-Smirnov test statistics for a goodness of fit were calculated in each distance bin *B _b_*. These were calculated under the null that


$$d_{ij}\in B_{b}\Rightarrow X_{ij}∼NB(s_{1}s_{2}f(d),r),$$

$$d∼U\left(d_{b}-\frac{w}{2},d_{b}+\frac{w}{2}\right),$$

where *w* is the width of the distance bin, and *s _1_*, *s _2_* are drawn from the distribution of the bait and other-end bias distributions. We implemented Monte Carlo hypothesis testing to obtain *p-*values, using 5,000 simulations of 5,000 pairs to measure the D-statistic, using the standard *p*-value estimator of Davison and Hinkley 1997 ^20^. Deriving a statistic per distance bin allowed us to examine how appropriate the null hypothesis is for separate distance bins. A cell-wise Bonferroni correction was applied to the significance threshold.

We plotted estimates of the distance function *f*( *d*) against the data, which is the geometric mean of the non-zero reads between bait-other end pairs in each distance bin, alongside a linear fit and a ‘theoretical’ fit. Specifically, we fit

- *logf*( *d*) = *a*_0_ + *a*_1_ *logd* + *a*_2_ ( *logd*) ^2^ + *a*_3_ ( *logd*) ^3^ (cubic)
- *logf*( *d*) = *b*_0_ + *b*_1_ *logd* (linear)
- *logf*( *d*) = *c*_0_ – *logd* (theoretical)

The ‘theoretical’ fit is of the form *f*( *d*) ∝ *d* ^–1^, as suggested to be the large-distance limit by Rosa *et al*. ^21^. We further calculated the integral of the curvature of *f* over the distances considered of [10 ^*4*^, *1.5* × 10 ^*6*^] base pairs, as a measure of the deviation from a power law, which would be represented by a straight line on a log-log scale. Specifically, we calculated *K*, given by


$$K=\int_{log\;1.0\times 10^{4}}^{log\;1.5\times 10^{6}}\kappa (x)\;dx,$$

$$\kappa =\frac{\left|y^{\prime \prime }\right|}{{\left(1+{y^{\prime }}^{2}\right)}^{\frac{3}{2}}},$$

$$y(x)=log\;f(e^{x}).$$

The limits of integration can be chosen as, outside of this range, the distance function is extrapolated linearly on a log-log scale, where the curvature *k* will be zero. Careful treatment of the second derivatives at these limits is not necessary. 

To validate the assumption that the technical noise will have minimal effect at small distance, the mean λ parameter for the pairs was calculated as per CHiCAGO. Moreover, boxplots were produced demonstrating the distribution of the parameter in each pool used in the estimation procedure.

To adjust for multiple testing in CHiCAGO, *p*-values are weighted according to the prior probability of a given null hypothesis being true or false ^9^. By default, weights are those estimated from human macrophage data, which defines reproducible interaction as one for which *log p* < –10 in all replicates. To evaluate this definition, we considered the function *g*( *ρ*) given by:


$$g(\rho )=\frac{\left|\left\{(i,j):score_{ij}<5\wedge log\;p_{ij}<\rho \right\}\right|}{\left|\left\{(i,j):log\;p_{ij}<\rho \right\}\right|}$$

For *ρ* = –10, *g* gives a FNR for reproducible interactions. Furthermore, we calculated the value of *ρ* that gives *g*( *ρ*) = 0.05 (assuming that such a value exists) to determine a *p*-value threshold for reproducible interactions coherent with the score statistic.

CHiCAGO’s algorithm is not symmetric in its treatment of bait-bait pairs and we found the correlation between non-zero values of *score _ij_* against *score _ji_* values for bait-bait pairs. By excluding pairs where both scores were zero, we avoided correlations being artificially inflated because there are many more non-interacting pairs than interacting pairs. We further computed the proportion of the bait-bait pairs that passed the advocated score threshold of > 5 in both pairs, relative to those passing the threshold in at least one pair, that is


$$\%\;\;reversible=\frac{\left|\left\{(i,j):score_{ij}>5\;or\;score_{ij}\;>5\right\}\right|}{\left|\left\{(i,j):score_{ij}>5\;and\;score_{ij}\;>5\right\}\right|}$$

This is equivalently the Jaccard index of the sets


$$\left\{(i,j):score_{ij}>5\right\}$$

and


$$\left\{(i,j):score_{ji}>5\right\}$$

where the Jaccard index of finite sets *A*_1_, *A*_2_, … , *A* *_K_* is


$$J(A_{1},A_{2},...,A_{K})=\frac{\left|\cap_{k=1}^{K}A_{k}\right|}{\left|\cup_{k=1}^{K}A_{k}\right|}$$

To examine the reproducibility of interactions called as significant by CHiCAGO, we produced alternate score thresholds based on: (i) a Bonferroni correction to control the FWER in the smallest distance bin; (ii) controlling for FNR, and (iii) minimising the FDR in Jaccard index between replicates. Specifically, for the Bonferroni correction, as thresholding at a score *α* requires that the evidence for an interaction exceeds that of a proximal pair with *p-*value *e* ^– *α*^, we imposed the threshold


$$\alpha =-log\frac{0.05}{\left|\left\{(i,j):d_{ij}\in B_{0}\right\}\right|}$$

The measure of reproducibility used was the Jaccard index of the sets of significant interactions in each replicate, which are reported as FDRs. Under the assumption that true interactions will be significant in all replicates and false interactions will not, we have the equivalence


FalseDiscoveryRate=1−JaccardIndex

At each score threshold, the number of interactions was reported to balance reliability and sensitivity. To demonstrate the importance in the choice of optimisation methodology in minimising FDR for a given cell line, two optimisation methods were used, Bound Limited-memory BFGS (L-BFGS-B), and the default NM utilised by optim in R.

We evaluated CHiCAGO’s weighting procedure. In the estimation of the weight parameters, CHiCAGO’s algorithm fits a monotonic decreasing curve to the observed prior-probability of interaction through bounded logistic regression. To examine the extent to which monotonicity is observed in the data, a lack-of-monotonicity statistic was generated for the data with the formula


$$LOM((v_{1},...,v_{n}))=1-\frac{\left|v_{n}-v_{1}\right|}{\sum_{i=1}^{n-1}\left|v_{i+1}-v_{i}\right|}$$

This is a natural formula in the sense that if the sequence ( *v*_*i*_) is monotonic, the lack of monotonicity is 0. To evaluate the fit we calculated a RSS; distance bins in which no interactions were observed were neglected, since for these bins CHiCAGO estimates the prior probability of interaction to be 0, hence log( *p*) is infinite. Thus, these points provide no information for the RSS, but nevertheless indicate a failure of the fitted model to represent the data, and so the presence of bins in which no interactions were observed is also reported.

We estimated the weight parameters at the threshold of –10 and *ρ*, for each cell line. Parameters that provided the ‘better’ fit (, *i.e.* had a lower RSS), were used and CHiCAGO re-run. After which, a Jaccard index was calculated for the sets of significant interactions called by CHiCAGO using either default or updated weight parameters.

All methods described were implemented in R version 3.6.3. A copy of the program, modified for readability above utility, is released as *Extended data* (Trim_of_CHiCAGO_evaluation.R) ^15^.

## Data availability

### Underlying data

ArrayExpress: Sequenced PCHi-C libraries for GM12878 cells. Accession number E-MTAB-2323: https://identifiers.org/arrayexpress:E-MTAB-2323

European Genome-phenome Archive (EGA): Sequenced PCHi-C libraries for ACD4, EP, Ery, FoeT, Mac0, Mac1, Mac2, MK, Mon, NACD4, NB, NCD4, NCD8, Neu, TB, TCD4 and TCD8 cells. Accession number EGAS00001001911: https://identifiers.org/ega.study:EGAS00001001911

European Genome-phenome Archive (EGA): Sequenced PCHi-C libraries for HT29 and LoVo cells. Accession number EGAS00001001946: https://identifiers.org/ega.study:EGAS00001001946

European Genome-phenome Archive (EGA): Sequenced PCHi-C libraries for KMS11 cells. Accession number EGAS00001002614: https://identifiers.org/ega.study:EGAS00001002614

European Genome-phenome Archive (EGA): Sequenced PCHi-C libraries for L428 cells. Accession number EGAS00001003032: https://identifiers.org/ega.study:EGAS00001003032

European Genome-phenome Archive (EGA): Sequenced PCHi-C libraries for NTERA2 cells. Accession number EGAS00001001930: https://identifiers.org/ega.study:EGAS00001001930

### Extended data

Zenodo: Algorithmic considerations when analysing capture Hi-C data. https://doi.org/10.5281/zenodo.4268400 ^15^.

This project contains the following extended data:

- Supplementary Figure 1.pptx (Distance function estimates for all cell lines. (a) ACD4; (b) EP; (c) Ery; (d) FoeT; (e) GM12878; (f) Mac0; (g) Mac1; (h) Mac2; (i) MK; (j) Mon; (k) NACD4; (l) NB; (m) NCD4; (n) NCD8; (o) Neu; (p) TB; (q) TCD4; (r) TCD8. Different coloured lines correspond to different models fitted: cubic (red), linear (blue) and theoretical of the form *f*( *d*) ∝ *d* ^–1^ as suggested to be the large-distance limit by Rosa *et al*. (green). Observed data is additionally marked.)
- Supplementary Figure 2.pptx (Technical Noise Estimates. (a) ACD4; (b) EP; (c) Ery; (d) FoeT; (e) GM12878; (f) Mac0; (g) Mac1; (h) Mac2; (i) MK; (j) Mon; (k) NACD4; (l) NB; (m) NCD4; (n) NCD8; (o) Neu; (p) TB; (q) TCD4; (r) TCD8. Boxplots showing the distribution of the technical noise parameter per pool.)
- Supplementary Figure 3.pptx (Plots of the score and its reverse for bait-bait pairs. (a) ACD4; (b) EP; (c) Ery; (d) FoeT; (e) GM12878; (f) Mac0; (g) Mac1; (h) Mac2; (i) MK; (j) Mon; (k) NACD4; (l) NB; (m) NCD4; (n) NCD8; (o) Neu; (p) TB; (q) TCD4; (r) TCD8. The five pairs with the largest deviation, (Score – Reverse Score) ^2^, are labelled with their IDs from the rmap file. The significance threshold of 5 is indicated by orange lines, with the blue line corresponding to the theoretical fit of Score=Reverse Score.)
- Supplementary Figure 4.pptx ( *p*-value weighting curve fit against prior probability of interaction. (a) ACD4; (b) EP; (c) Ery; (d) FoeT; (e) GM12878; (f) Mac0; (g) Mac1; (h) Mac2; (i) MK; (j) Mon; (k) NACD4; (l) NB; (m) NCD4; (n) NCD8; (o) Neu; (p) TB; (q) TCD4; (r) TCD8.)
- Supplementary Figure 5.pptx (Distance function estimates for all cancer cell lines. (a) BLN2; (b) BLN3; (c) HT29; (d) KMS11; (e) KMS12BM; (f) L428; (g) LoVo; (h) MM1S; (i) N16; (j) NTERA2. Different coloured lines correspond to different models fitted: cubic (red), linear (blue) and theoretical of the form *f*( *d*) ∝ *d* ^–1^ as suggested to be the large-distance limit by Rosa *et al.* (green). Observed data is additionally marked.)
- Supplementary Figure 6.pptx (Plots of the score and its reverse for bait-bait pairs for cancer cell lines. (a) BLN2; (b) BLN3; (c) HT29; (d) KMS11; (e) KMS12BM; (f) L428; (g) LoVo; (h) MM1S; (i) N16; (j) NTERA2. The five pairs with the largest deviation, (Score – Reverse Score) ^2^, are labelled with their IDs from the rmap file. The significance threshold of 5 is indicated by orange lines, with the blue line corresponding to the theoretical fit of Score=Reverse Score.)
- Supplementary Figure 7.pptx (Identification of large-scale chromosomal translations from Heatmaps of Hi-C contacts. (a) BLN2; (b) BLN3; (c) HT29; (d) ACD4; (e) L428. Lighter regions correspond to interactions with reads greater than background levels, with the diagonal representing the reads expected by Brownian motion in a karyotypically normal cell.)
- Supplementary Table 1.docx (PCHi-C datasets analysed. Indicated for each cell type is the number of replicates analysed, the Hi-C library preparation method (dilution or in situ) and the set of baits used for promoter capture (V1, original or V2, updated).)
- Supplementary Table 2.docx (Discrete KS test applied to the null distribution. Bin-wise D statistics and the corresponding Monte Carlo *p*-value for all non-cancer cell lines.)
- Supplementary Table 3.docx (Coefficients of the corresponding fits of the distance function *f*( *d*). Coefficients fitted on a log-log scale over data in the range 1.0 × 10 ^4^ ≤ *d* ≤ 1.5 × 10 ^6^, *d* given in base pairs.)
- Supplementary Table 4.docx (Coefficients of the corresponding fits of the distance function for cancer cell lines. Coefficients fitted on a log-log scale over data in the range 1.0 × 10 ^4^ ≤ *d* ≤ 1.5 × 10 ^6^, *d* given in base pairs.)
- Supplementary Data 1.xlsx (Read counts for PCHi-C datasets at stages of HiCUP processing.)
- Trim_of_CHiCAGO_evaluation.R (The main Rscript used to run this analysis, containing an implementation of the methods described)

Data are available under the terms of the Creative Commons Attribution 4.0 International license (CC-BY 4.0).
