# Supplementary material for: Algorithmic considerations when analysing capture Hi-C data
Source: Wellcome Open Res. 2022 Nov 11;5:289. Originally published 2020 Dec 14. [Version 2] doi: 10.12688/wellcomeopenres.16394.2 (PMC9699993; doi:10.12688/wellcomeopenres.16394.2)
Supplement: Peer review history

# Peer review: Reviewer response for version 2

**DOI:** 10.21956/wellcomeopenres.20567.r53275

**Published:** 2022-11-25

**Authors:** Mikhail Spivakov

**Affiliations:** 1 MRC London Institute of Medical Sciences, London, UK; 2 Imperial College London, London, UK

I am happy with the revisions made.

Is the rationale for developing the new method (or application) clearly explained?

Yes

Is the description of the method technically sound?

Yes

Are the conclusions about the method and its performance adequately supported by the findings presented in the article?

Yes

If any results are presented, are all the source data underlying the results available to ensure full reproducibility?

Yes

Are sufficient details provided to allow replication of the method development and its use by others?

Yes

Reviewer Expertise:

Developer of CHiCAGO pipeline

I confirm that I have read this submission and believe that I have an appropriate level of expertise to confirm that it is of an acceptable scientific standard.

# Peer review: Reviewer response for version 1

**DOI:** 10.21956/wellcomeopenres.18034.r46160

**Published:** 2021-09-29

**Authors:** Mikhail Spivakov

**Affiliations:** 1 MRC London Institute of Medical Sciences, London, UK; 2 Imperial College London, London, UK

Disney-Hogg *et al.* evaluate the performance of CHiCAGO, a pipeline for signal detection in Capture Hi-C data, with respect to model fit, multiple testing treatment and the choice of significance threshold. The paper highlights the importance on training *p*-value weights on own data and the challenges of choosing an optimal score threshold in the absence of a gold standard. 

 As a developer of CHiCAGO, I thank the authors for an independent evaluation of our pipeline and am happy to see that it has withstood most tests.

 I only have a very small number of comments:

1. "In most cell lines the *p*-values associated with the small distance bins were effectively zero but rapidly increased to near-unity in the larger distance bins". In the authors' opinion, why are the p-values so low in the small distance bins? It would be good to state this more explicitly. 
2. It would be good to unpick a bit more the phenomena that underlie "non-reversible" bait-to-bait contacts - which are generally expected due to the asymmetrical nature of CHi-C data and CHiCAGO's analytical approach. Theoretically, this may be to do with challenges in estimating s_i's (other-end scaling factors) based on much less information than available for s_j's (bait scaling factors). Secondly, this could be due to differences in the coverage of respective baits, potentially leading to differential sensitivity in signal detection depending on the viewpoint. Finally, from Fig 2 it seems that there are a few cases where the difference in score is quite small and so their position on either side of the threshold is incidental.
  It might be worth discussing these possible situations a bit more detail. Also, what is the authors' suggested strategy for dealing with these situations? In our lab, we pick the pair with the higher score, as we believe that false-negatives are a generally bigger issue in CHi-C and CHiCAGO than false-positives. Alternatively, bait-to-bait contacts could be analysed separately as a symmetrical matrix using appropriate tools.
3. I wonder to what extent the score threshold selection methods based on reproducibility across replicates are inflated due to sparsity issues (as we showed in Cairns *et al.*, 2016, Fig S4). ^1^ This is the reason why in Javierre ^2^ we used a different approach (based on Blangiardo *et al.*) ^3^ to show *consistency* between datasets, while in Freire-Pritchett eLife 2017; Nat Prot 2021 ^4^^,^^5^ use a threshold-tuning approach based on balancing enrichment and recall of PIRs containing enhancer-associated histone marks. It would be good to discuss these points.

Is the rationale for developing the new method (or application) clearly explained?

Yes

Is the description of the method technically sound?

Yes

Are the conclusions about the method and its performance adequately supported by the findings presented in the article?

Yes

If any results are presented, are all the source data underlying the results available to ensure full reproducibility?

Yes

Are sufficient details provided to allow replication of the method development and its use by others?

Yes

Reviewer Expertise:

Developer of CHiCAGO pipeline

I confirm that I have read this submission and believe that I have an appropriate level of expertise to confirm that it is of an acceptable scientific standard, however I have significant reservations, as outlined above.

## References

[1] : CHiCAGO: robust detection of DNA looping interactions in Capture Hi-C data. *Genome Biology* .2016;17(1) : 10.1186/s13059-016-0992-2 10.1186/s13059-016-0992-2 27306882PMC4908757

[2] : Lineage-Specific Genome Architecture Links Enhancers and Non-coding Disease Variants to Target Gene Promoters. *Cell* .2016;167(5) : 10.1016/j.cell.2016.09.037 1369-1384.e19 10.1016/j.cell.2016.09.037 PMC512389727863249

[3] : sdef: an R package to synthesize lists of significant features in related experiments. *BMC Bioinformatics* .2010;11: 10.1186/1471-2105-11-270 270 10.1186/1471-2105-11-270 20487547PMC3239329

[4] : Global reorganisation of cis-regulatory units upon lineage commitment of human embryonic stem cells. *eLife* .2017;6: 10.7554/eLife.21926 10.7554/eLife.21926 PMC540786028332981

[5] : Detecting chromosomal interactions in Capture Hi-C data with CHiCAGO and companion tools. *Nat Protoc* .2021;16(9) : 10.1038/s41596-021-00567-5 4144-4176 10.1038/s41596-021-00567-5 34373652PMC7612634

# Response

**Published:** 2022-11-04

**Authors:** Linden Disney-Hogg

Thank you for providing us with the opportunity to submit a revised manuscipt. I apologise for the delay in responding, however this unfortunate delay was the consequence of me starting a PhD on an unrelated subject compounded by Covid-related issues. All your comments were extremely insightful and pertinent, and we have largely implemented all the changes or additions highlighted, with only two minor exceptions. These we detail below.

1. Change made, with the reason being that the model assumptions which give a negative binomial distribution breaking down at small distances.
2. We discuss the asymmetry and approaches further, not providing a fixed strategy used by the authors, but noting what considerations one might make depending on the goal of the Hi-C investigation, as well as future approaches to consider including those highlighted by the reviewer.
3. We mention the possible skew effect of sparsity, which we expect to be small, and comment on the possible link to comment 9 of Reviewer 1.

These changes you will see in an updated version of the paper. Having made changes to the manuscript we hope it is now suitable for indexing.

# Peer review: Reviewer response for version 1

**DOI:** 10.21956/wellcomeopenres.18034.r42479

**Published:** 2021-02-18

**Authors:** Borbala Mifsud

**Affiliations:** 1 College of Health and Life Sciences, Hamad Bin Khalifa University, Doha, Qatar

Disney-Hogg *et al. *have considered what the important features of algorithms used for identifying biologically meaningful interactions in capture Hi-C analysis are. They used CHiCAGO, a widely applied algorithm, as their case study and assessed the appropriateness of the distance function used for correction, the effect of altering the weights in the model, looked at how thresholding influences the resulting significant interactions and analysed the reciprocity of bait-bait interactions. Given the lack of a set of gold standard interactions, this evaluation is based on the assumption that true interactions are reproducible.

 They performed the analyses both in normal hematopoietic cells and in cancer cell lines. They found that the cubic spline fits the data well and that the model used by CHiCAGO creates highly asymmetric results for bait-bait interactions. Changing the weights to custom calculated ones slightly improves the quality of the identified interactions. The suggested threshold for significance is appropriate for most cell lines to optimize FNR and FDR, however, there are notable exceptions to this.

 There are a number of methods developed for capture Hi-C data analysis and this approach can be applied to evaluate and compare their performance and therefore it is a valuable contribution to the field.

**Minor comments:**

1. Promoter is the correct term for the genomic region instead of promotor.
2. Supplementary data 1 is missing information on the captured read count.
3. In figure 1, the fragmentation step is missing after the ligation “Fragmentation, biotin pull-down, adapter ligation and PCR”.
4. Table 1 would probably be easier to see in a plot where the test statistics and the p-value are plotted against the distance.
5. Please add labels to the scales in Supp. Figure 2.
6. In figures where the distance is one of the variables, could you indicate the unit?
7. The definition of FDR based on Jaccard distance should be in the main text.
8. In Table 2, it would add valuable information if the number of significant interactions were included for each threshold.
9. Could you comment on the observation that the optimal threshold for FDR using BFGS was mostly around the recommended threshold, but in some cases it was very different? Do those data sets share any characteristics that could explain it?

Is the rationale for developing the new method (or application) clearly explained?

Yes

Is the description of the method technically sound?

Yes

Are the conclusions about the method and its performance adequately supported by the findings presented in the article?

Yes

If any results are presented, are all the source data underlying the results available to ensure full reproducibility?

Yes

Are sufficient details provided to allow replication of the method development and its use by others?

Yes

Reviewer Expertise:

Epigenomics, 3D chromatin organization

I confirm that I have read this submission and believe that I have an appropriate level of expertise to confirm that it is of an acceptable scientific standard.

# Response

**Published:** 2022-11-04

**Authors:** Linden Disney-Hogg

Thank you for providing us with the opportunity to submit a revised manuscript. I apologise for the delay in responding, however this unfortunate delay was the consequence of me starting a PhD on an unrelated subject compounded by Covid-related issues. All your comments were extremely insightful and pertinent, and we have largely implemented all the changes or additions highlighted, with only two minor exceptions. These we detail below.

1. Change made.
2. We note that the missing data is available upon request.
3. Error rectified.
4. We would suggest that actually there is not much to be gained from plotting the data, as the p-value plot would be effectively a step function, and this qualitative information is just as easily gained from a table.
5. Change made.
6. We highlight that the distances were measured in base-pairs, but in fact as we never plot distance itself, only log(distance), a change of units would correspond to a horizontal translation of the data. This degree of freedom is fully captured by the parameters of the fitting models in each case, and so has no biological significance.
7. Change made.
8. Change made for NST and the FWER SST.
9. Change made, but no explaining characteristics found.

These changes you will see in an updated version of the paper. Having made changes to the manuscript we hope it is now suitable for indexing.
